# Why the Orientational Mobility in Arginine and Lysine Spacers of Peptide Dendrimers Designed for Gene Delivery Is Different?

**DOI:** 10.3390/ijms21249749

**Published:** 2020-12-21

**Authors:** Valeriy V. Bezrodnyi, Oleg V. Shavykin, Sofia E. Mikhtaniuk, Igor M. Neelov, Nadezhda N. Sheveleva, Denis A. Markelov

**Affiliations:** 1 St. Petersburg State University, 7/9 Universitetskaya nab., 199034 St. Petersburg, Russia; v.v.bezrodniy@mail.ru (V.V.B.); shevelevann@gmail.com (N.N.S.); 2 Faculty of Applied Optics and Bioengineering Institute, St. Petersburg National Research University of Information Technologies, Mechanics and Optics (ITMO University), Kronverkskiy pr. 49, 197101 St. Petersburg, Russia; mikhtanyuk@mail.ru (S.E.M.); i.neelov@mail.ru (I.M.N.)

**Keywords:** peptide dendrimer, computer simulation, molecular dynamics, zeta potential, NMR, spin-lattice relaxation time

## Abstract

New peptide dendrimer with Lys-2Arg repeating units was recently studied experimentally by NMR (RSC Advances, 2019, 9, 18018) and tested as gene carrier successfully (Int. J. Mol. Sci., 2020, 21, 3138). The unusual slowing down of the orientational mobility of 2Arg spacers in this dendrimer was revealed. It has been suggested that this unexpected behavior is caused by the Arg-Arg pairing effect in water, which leads to entanglements between dendrimer branches. In this paper, we determine the reason for this slowing down using atomistic molecular dynamics simulation of this dendrimer. We present that the structural properties of Lys-2Arg dendrimer are close to those of the Lys-2Lys dendrimer at all temperatures (Polymers, 2020, 12, 1657). However, the orientational mobility of the H-H vector in CH${}_{2}$-N groups of 2Arg spacers in Lys-2Arg dendrimer is significantly slower than the mobility of the same vector in the Lys-2Lys dendrimer. This result is in agreement with the recent NMR experiments for the same systems. We revealed that this difference is not due to the arginine-arginine pairing, but is due to the semiflexibility effect associated with the different contour length from CH${}_{2}$-N group to the end of the side arginine or lysine segment in spacers.

## 1. Introduction

Dendrimers are regular polymers branched from a single core. Their repeating units have a fork-like structure, they usually have two (rarely three or more) prongs [1,2,3]. For these reasons, the number of atoms in each next generation is doubled (or tripled), and the dendrimers have an almost spherical shape and many terminal groups available for functionalization. These structural features of dendrimers have led to their widespread use in industry and biomedicine [4,5]. The most popular dendrimers are polyamidoamine (PAMAM), peptide, polypropyleneimine (PPI)) and carbosilane [6,7,8,9]. One of the most important applications of dendrimers in medicine is their use as carriers of drugs [10] and genetic material [11] for gene therapy [12,13]. Gene delivery can be performed using viral [14] or non-viral [15] carriers. The first non-viral carriers were linear cationic homopolymers, block-copolymers [16] and liposomes [17] based on cationic lipids. Cationic polysaccharides [18] and cationic peptides [19,20] were also used for this purpose. In particular, peptides and other polymers enriched with positively charged aminoacid residues such as lysine [21,22], arginine [18], and histidine [23] are popular delivery vehicles.

Synthetic dendrimers (PAMAM, PPI, and others) with positively charged groups have been used in gene delivery for a long time [24,25,26]. To improve gene delivery properties, the terminal groups of dendrimers were sometimes functionalized with charged aminoacid residues, for instance with lysine, arginine and histidine [27]. Lysine dendrimers consisting only of lysine aminoacid residues were used for this purpose as well [28,29,30,31,32,33,34]. Lysine-based dendrimers with terminal lysine residues replaced to arginine or histidine aminoacid residues were also tested as drug and gene carriers [35,36,37,38]. Lysine dendrimers with short terminal peptides were used as multiple antigen peptides (MAPs) [39].

The experimental study of the size of lysine dendrimers in dimethylformamide solvent was carried out in the early 1980s [40,41]. Later, the properties of these dendrimers were studied in water [31,42,43]. The molecular dynamics (MD) simulations of lysine dendrimers have been performed [43,44,45,46,47]. The structure and mobility of lysine dendrimers of different generations have been studied.

Peptide dendrimers were synthesized shortly after the synthesis of lysine dendrimers. Unlike lysine dendrimers, they can consist of any aminoacid residues. More often one, two, or three amino acid residues or short peptide fragments are attached to the terminal groups [35,36,37,38,39] or sometimes they are inserted between neighboring branching points as spacers in regular lysine dendrimers [39,42,48,49,50,51] or even in the dendrimer core.

Recently new lysine-based peptide dendrimers with repeating units Lys-2Lys, Lys-2Gly, Lys-2Arg, Lys-2His, which consist of lysine branching points and dipeptide spacers (2Lys, 2Gly, 2Arg, 2His) between them have been synthesized and studied by NMR [52,53,54]. The unexpected slowing down of the orientational mobility of the side CH${}_{2}$ groups in 2Arg spacers [53] was found. Applications of these dendrimers for siRNA delivery have been studied as well [55,56]. It was shown that the Lys-2Arg dendrimer is better carrier of genetic material than the Lys-2Gly or Lys-2Lys dendrimers. The authors of the paper [53] suggested that the slowing down of the mobility of the side groups in spacers of Lys-2Arg in comparison with Lys-2Lys is due to the arginine-arginine pairing effect (which is well known for linear arginine peptides [57,58]).

In the literature there are examples of computer simulation of peptide dendrimers using MD [59,60,61], Brownian dynamics (BD) [62,63,64] and numerical self-consistent field (SCF) methods [65,66,67].

The main goal of this work is to perform MD simulation of the Lys-2Arg dendrimer to study its structural and dynamical properties. We compare the simulation data with the results of the NMR study of this dendrimer [53] and with simulation [61] and NMR results [52] for the Lys-2Lys dendrimer to understand the reason for the experimentally observed difference in the mobility of side groups of 2Arg and 2Lys spacers in the Lys-2Lys and Lys-2Arg dendrimers which have the same backbone and similar distribution of charges along it [52,53,54].

## 2. Materials and Methods

**Figure 1:**
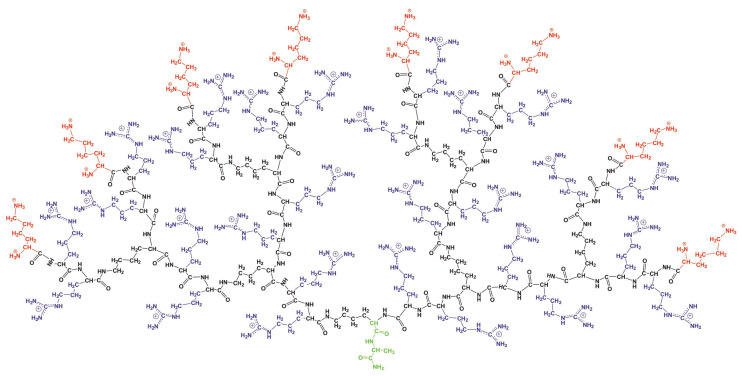
The chemical structure of the Lys-2Arg dendrimer. The dendrimer core is marked by green color, the backbone by black color, the side Arg segments by violet color and the terminal lysine segments by red color.

The peptide dendrimer of the second-generation with Lys-2Arg repeating units was studied by the molecular dynamics method. We used the full atomic model of this dendrimer shown in Figure 1. The characteristics of the Lys-2Arg dendrimer are provided in Table 1. The Lys-2Arg dendrimer has an alanine-lysine core (marked by green color in Figure 1), a backbone formed by the main peptide chain of Lys-2Arg repeating units (black), and terminal lysine segments (red). The number of repeating units $N_{ins}$ is equal to 28 and the number of terminal NH${}_{3}^{+}$ groups $N_{end}$ is 16. The bulky charged side segments of 2Arg spacers are marked by violet. The cationic Lys-2Arg was placed in a cubic box with periodical boundary conditions filled by water and Cl-counterions. The bare charge of the dendrimer ($Q_{bare}=+44$) consists of the sum of the charge of terminal lysine groups ($Q_{end}=+16$) and the charge of 2Arg spacers ($Q_{ins}=+28$). The number of counterions is equal to 44 (see Table 1).

The Gromacs package [68] and the AMBER-99SB-ILDN force field [69] were used in all molecular dynamics simulations. We have used the computer programs to calculate the characteristics of our dendrimer as described in our previous papers on simulation of linear polymers and polyelectrolytes, polymers brushes [70], AFM of linear biopolymers [71], dendrimers and hyperbranched polymers in shear and elongational flow [72,73] and cyclization of linear peptides [74].

The preparation for the simulation consisted of several stages including optimization of the initial molecular structure without solvent and its equilibration in water using several MD simulations runs with different timesteps. This preparation is described in detail [61]. The final MD simulations were carried out with an integration step of 1 fs to get the productive trajectory. The NPT ensemble was implemented via the Nose-Hoover thermostat (at constant temperatures of 280, 290, 300, 310, 320 and 340 K) with the time constant τ = 0.4 ps [75] and the Parrinello-Rahman barostat [76] with τ = 0.5 ps and with the compressibility of water, weakly dependent on temperature [77]. Four sequential 250 ns simulation runs (1000 ns in total) were carried out at each temperature. The coordinates of the system were saved to a file every 100 fs for further processing.

## 3. Results

### 3.1. The Global Characteristics

To characterize the statistical and dynamical behavior of the dendrimer as a whole we calculated its size and shape as well as the time correlation functions of the size fluctuation and the rotation as a whole. The mean-squared radius of gyration $R_{g}$ is one of the parameters for estimating the characteristic size of the dendrimer. We can obtain $R_{g}$ using static light scattering, small angle neutron scattering and small angle X-ray scattering. In simulation $R_{g}$ can be calculated as

(1) $$R_{g}=\sqrt{\frac{1}{M}\sum_{i}m_{i}r_{i}^{2}},$$

where $M,\;m_{i}$ are the molecular masses of the dendrimer and its *i*-th atom, correspondingly, and $r_{i}$ is the distance from the *i*-th atom to the center of mass of the dendrimer. In accordance with the physical meaning of $R_{g}$ (Equation (1)), it provides information about the distribution of the mass of a dendrimer around its center of mass (the larger $R_{g}$, the lower the rotation time of the dendrimer as a whole).

**Figure 2:**
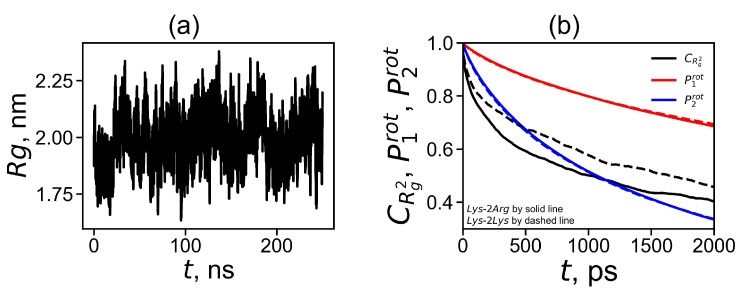
(**a**) The time evolution of the mean-squared gyration radius $R_{g}$ for the Lys-2Arg dendrimer at T=310 K, (**b**) the autocorrelation functions (ACFs) for Lys-2Arg and Lys-2Lys: the orientational ACFs $P_{1}^{rot}\left(t\right)$ and $P_{2}^{rot}\left(t\right)$ for the core-to-end vector and the ACF for the pulsation of $R_{g}^{2}$ values at temperature T=310 K.

Equation (1) was used to calculate the instant size of the dendrimer in each time moment (saved every 100 ps in corresponding frames of 1000 ns trajectory file). The time dependence of the radius of gyration shows the pulsation of the dendrimer size (see Figure 2a). It can be seen from that the size fluctuates between 1.7 nm and 2.3 nm. These fluctuations practically do not depend on temperature (not shown) and are similar to those of the Lys-2Lys dendrimer [61].

The fluctuations in the radius of gyration indicate that the dendrimer is not a rigid spherical object with a constant radius, but is a molecule with a radius that pulsate in a rather wide range. This pulsating process can be described using the time autocorrelation function that characterizes the correlation of dendrimer sizes

(2) $$C_{R_{g}^{2}}\left(t\right)=\frac{\langle R_{g}^{2}\left(\tau \right)\cdot R_{g}^{2}\left(\tau +t\right)\rangle -{\langle R_{g}^{2}\rangle }^{2}}{\langle R_{g}^{4}\rangle -{\langle R_{g}^{2}\rangle }^{2}}$$

The comparison of the functions $C_{R_{g}^{2}}\left(t\right)$ for Lys-2Arg (solid black curve) and for Lys-2Lys (dashed black curve) is shown in Figure 2b. It is easy to see that this function for Lys-2Arg decreases with time slower than for the Lys-2Lys dendrimer. We will discuss this question in more detail later.

The rotation mobility of the dendrimer can be estimated using the first-order orientational autocorrelation function (ACF)

(3) $$P_{1}^{rot}\left(t\right)=\langle \frac{r(t)\cdot r(0)}{\left|r(t)\right|\left|r(0)\right|}\rangle$$

and the second-order orientational ACF

(4) $$P_{2}^{rot}\left(t\right)=\frac{3}{2}\langle \frac{{\left(r(t)\cdot r(0)\right)}^{2}}{{\left|r(t)\right|}^{2}{\left|r(0)\right|}^{2}}\rangle -\frac{1}{2}$$

of the core-to-end vector (the vectors r(t) connect the first branching point in the core of the dendrimer and the C atoms of its terminal NH${}_{3}^{+}$ groups). To obtain better statistical results we calculated the orientational ACFs (Equations (3) and (4)) for each of sixteen r(t) vectors (to each terminal group). The averaged time dependencies of these ACFs for Lys-2Arg and Lys-2Lys are shown in Figure 2b. We can see the similar behavior of these curves.

The rotation time of the dendrimer as a whole can be estimated as time $\tau_{rot}^{P_{1}}$ where the function $P_{1}^{rot}\left(t\right)$ decays in *e* times. The rotation times $\tau_{rot}^{P_{1}}$ obtained for the dendrimer as a whole are presented in Table 2. For both dendrimers, the rotation time decreases with increasing temperature. The difference in the rotation times of these two dendrimers does not exceed 10 percent, which is close to the calculation error of this value. This means that the global rotational motion of the dendrimer as a whole is very similar for both dendrimers.

For discussion of the rotation of the dendrimer as a whole, it is important to know the shape of the rotating object. The shape of the object is also important in practical applications. For example, it is well known that the penetration of rod-shaped molecules and spherical molecules through cell membranes is different [78]. Many simulation works have shown that the dendrimers of the small generations are asymmetric, but become more spherical as the number of generations increases [79,80,81]. The dendrimer shape can be evaluated using the asphericity parameter α according to the following formula [80,82,83]

(5) $$\alpha =1-3\frac{I_{x}I_{y}+I_{x}I_{z}+I_{y}I_{z}}{{\left(I_{x}+I_{y}+I_{z}\right)}^{2}}$$

where values $I_{x}$, $I_{y}$, $I_{z}$ are the eigenvalues of the gyration tensor which are equivalent to ellipsoid axes of a prolate or oblate molecule. In the case of a very prolate molecule, for example, for a rod-like molecule, one axis dominates over the others, and the asphericity in this limiting case tends to unity, while for a spherical molecule α is close to zero. Table 3 shows the value of α for the Lys-2Lys and Lys-2Arg dendrimers obtained from MD simulation at T=310 K.

**Figure 3:**
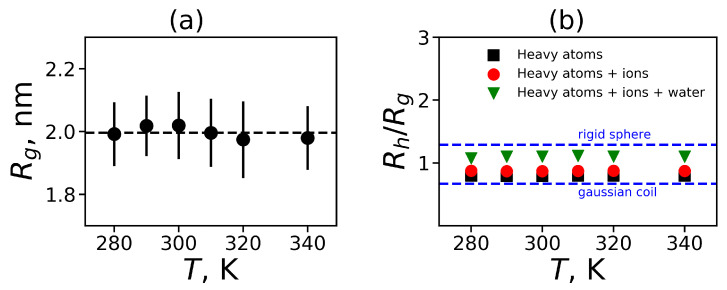
The global characteristics for Lys-2Arg as functions of temperature: (**a**) the mean-squared radius of gyration $R_{g}$, (**b**) the characteristic ratio $R_{h}$ (in the Kirkwood approximation) to the radius of gyration $R_{g}$ for Lys-2Arg for three alternative ways of calculations: taking into account the heavy atoms of this dendrimer only, for heavy atoms in the dendrimer and ions, for all heavy atoms in the system (carbons, nitrogens, and oxygens of the dendrimer; Cl ions; oxygens of water molecules).

We obtained that the value of α for both dendrimers is equal to 0.02, which is very close to 0. It means that we can consider our dendrimers as spherical molecules. We have used the radius of gyration $R_{g}$ to characterize the spherical molecule. The temperature dependence of $R_{g}$ is presented in Figure 3a. Also, this figure shows the standard deviations from the equilibrium size in the form of errorbars. It can be seen that the dendrimer size $R_{g}$ is practically independent of temperature.

The hydrodynamic radius $R_{h}$ is another characteristic of the molecule size that can be measured experimentally. The hydrodynamic radius $R_{h}$ is usually estimated as the Stokes radius, i.e., it can be calculated using the coefficient of translational diffusion of the center of mass of the dendrimer. Here, we estimated the hydrodynamic radius of the dendrimer from MD simulation using the Kirkwood approximation [84,85]:

(6) $$R_{h}^{-1}={\langle r_{ij}^{-1}\rangle }_{i\neq j},$$

where $r_{ij}$ is a distance between two atoms *i* and *j*. The proposed formula can be utilized in several ways. In some works on MD simulation of peptides and proteins only C${}_{\alpha }$ carbon atoms of the main peptide chain are used. Other methods take into account all heavy atoms in the backbone of a peptide or peptide dendrimer. In addition, all heavy dendrimer atoms and ions or even all heavy dendrimer atoms, ions and oxygen in water molecules that are close to the dendrimer atoms can be considered. We used all of these approaches and found the characteristic ratio $R_{h}/R_{g}$ for the Lys-2Arg dendrimer as a function of temperature (Figure 3b). In Figure 3b we also indicate two theoretical limits—for a penetrable gaussian coil and for an unpenetrable rigid sphere [86]. In all cases the obtained MD data are between two limiting values for these theoretical models. An increase in the number of types of heavy atoms that are taken into account when calculating the $R_{h}$ using Equation (6) leads to a better description of the MD data by the unpenetrable sphere model (upper dashed line).

In addition to $R_{g}$ and $R_{h}$, it is important to estimate the position of the outer boundary of the dendrimer in the solvent (i.e., the radial distance between the center of the dendrimer and its spherical surface). This parameter provides a good estimation of the size of the dendrimer as a nanocontainer.

The position of the outer boundary of the sphere can be calculated theoretically as $\sqrt{5/3}R_{g}$ or through the estimation of the position of the terminal groups $R_{e}$. We can calculate $R_{e}$ as the mean square radial distance from the center of the dendrimer to the N atoms at the terminal NH${}_{3}^{+}$ groups:

(7) $$R_{e}={\left(\frac{1}{N_{t}}\sum_{i=1}^{N_{t}}r_{i}^{2}\right)}^{1/2},$$

Moreover, in simulation, we can use the effective radius $R_{max}$ of the dendrimer as a charged macroion. $R_{max}$ is estimated from the position of the slip plane and will be determined below from the electrostatic properties of the dendrimer. All of these characteristics ($\sqrt{5/3}R_{g}$, $R_{e}$, $R_{max}$) are given in Table 3. It is interesting to note that the value of $R_{e}$ is close to the theoretical value of the radius of the rigid sphere equal to $\sqrt{5/3}R_{g}$.

### 3.2. The Local Structure

#### 3.2.1. The Spatial Symmetry and Atomic Distributions

**Figure 4:**
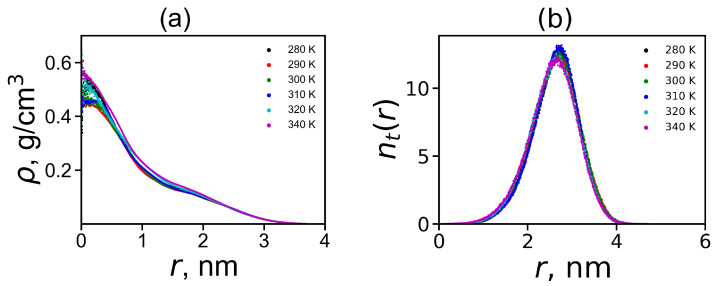
The radial density distribution function of the Lys-2Arg dendrimer atoms (**a**) and the radial distribution function of the number of terminal atoms (**b**) at different temperatures. Both functions are counted from the center of mass of the dendrimer.

The local structure of the spherical molecule is described by the density profile, i.e., by the radial distribution of the atomic density ρ(r):

(8) $$\rho \left(r\right)=\frac{1}{4\pi r^{2}}\sum_{i}m_{i}\delta \left(r-r_{i}\right)$$

where δ is the Dirac delta function, *r* is the radial distance from the center of mass of the molecule, $r_{i}$ is the radial distance from *i*-th atom to the center of mass of the molecule. Figure 4a shows the radial distributions ρ(r) for Lys-2Arg dendrimer at different temperatures. In this figure all curves practically coincide. Four main areas can be distinguished on the density profile: (1) the plateau-like region in the center at 0<r<0.5; (2) the fast decay at 0.5<r<1.0; (3) the second plateau-like region at 1.0<r<2.0; 4) the second fast decay at r>2.0. The radial density ρ(r) is always higher near the center of mass of the dendrimer (about r=0) and decreases with increasing radial distance. These results are very similar to the results for the Lys-2Lys dendrimer with the same backbone and similar distribution of charges along it.

The radial distribution of the number of terminal groups $n_{t}\left(r\right)$ of the Lys-2Arg dendrimer demonstrates whether the terminal groups can bend backward and penetrate into the dendrimer interior (the back-folding effect) or not. The results shown in Figure 4b indicate that the backfolding occurs. This is similar to the backfolding for the Lys-2Lys dendrimer but less than in the usual lysine dendrimer with single Lys aminoacid residue in repeating units [47]. This is because both the Lys-2Arg and Lys-2Lys dendrimers have additional charged groups in 2Arg or 2Lys spacers. The curves are practically independent of temperature and have an almost symmetrical shape (with a slightly longer tail at short distances *r*) with a maximum at a distance of around 2.6.

The radial distribution functions ρ(r) and $n_{t}\left(r\right)$ have a coordinate *r*, which is the radial distance from the center of mass of the dendrimer. However, in the spherical coordinate system there are two other coordinates, ϕ and ψ angles. If the dendrimer atoms are not evenly distributed at given *r* (i.e., density is not the same at different ϕ and ψ), then the dendrimer has not quite a homogeneous structure. Such heterogeneity can arise, for example, if the dendrimer in aqueous solution has hydrophobic groups or other groups tend to associate. This property is not expected for the dendrimer under study with positively charged terminal groups (and spacer) that repel each other. However, we calculated the congregation coefficient $k_{45}$ [44] which characterizes the possible inhomogeneity of the distribution of atoms in different spherical sectors. It is known that for the strongly associated terminal groups concentrated in one sector, this parameter is equal to 1. In the case of uniform scattering of terminal groups over all spherical sectors, this parameter is close to zero. The calculated values of this parameter for the terminal groups of the Lys-2Arg and Lys-2Lys dendrimers are presented in Table 3. The congregation coefficient $k_{45}$ for these groups is equal to 0.1. This means that the terminal groups are distributed fairly evenly in both dendrimers.

#### 3.2.2. Electrostatic Interactions

**Figure 5:**
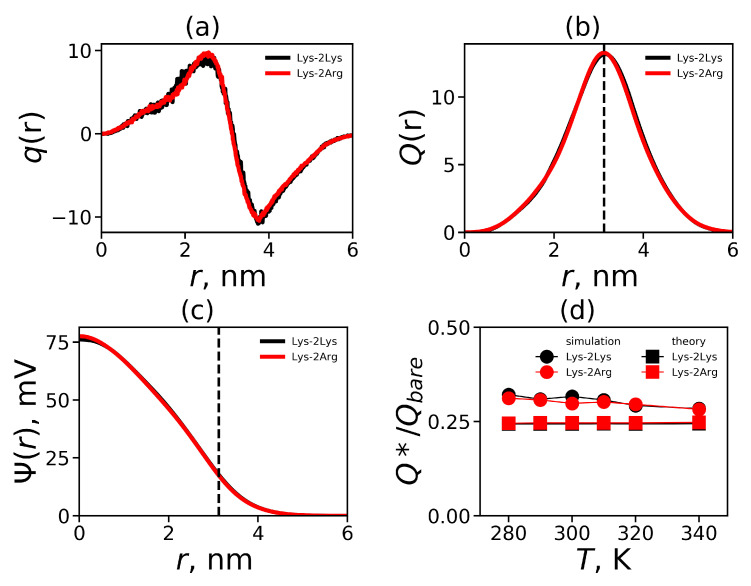
(**a**) The total charge distribution q(r), (**b**) the cumulative charge distribution Q(r), (**c**) the electrostatic potential Ψ(r) for Lys-2Arg and Lys-2Lys at T=310 K; (**d**) the temperature dependencies of the relative effective charge $Q^{*}/Q_{bare}$ for Lys-2Arg.

One of the most important properties of electrostatic interactions in a dendrimer is the distribution of the total charge q(r) relative to the center of mass of the dendrimer. We show an example of this distribution for Lys-2Arg dendrimer obtained by MD simulation at temperature T=310 K (by red color) in Figure 5a. For comparison, the curve for Lys-2Lys, which has a similar distribution of charges along the dendrimer backbone, is also presented (by black color). This figure clearly shows that the distributions for two dendrimers practically coincide. Furthermore, the total charge distribution curve q(r) includes the positive part with the maximum at r=2.6 and the negative part with the minimum at r=3.6. The total charge distribution of this shape is often referred to as the charge distribution of the electrical double layer.

From the total charge distribution q(r), you can calculate the cumulative charge Q(r), which is an integral characteristic. Figure 5b illustrates the cumulative charge distribution for the Lys-2Arg and Lys-2Lys dendrimers. We show the position of the maximum $R_{max}$ (see Table 3) by the vertical dashed line. The maximum corresponds to the value of the effective (uncompensated) charge $Q^{*}$ of the dendrimer (see Table 4). The surface charge density $\sigma =Q^{*}/4\pi R_{max}^{2}$ [87] and the degree of a charge renormalization of the dendrimer $Q^{*}/Q_{bare}$ can be calculated as well (see Table 4).

Electrostatic interactions are long-range, and characteristic lengths are used to describe them. One of these characteristics is the Bjerrum length:

(9) $$\lambda_{B}=\frac{e^{2}}{4\pi \epsilon \epsilon_{0}k_{B}T}$$

where *e* is the elementary charge, ϵ is the relative dielectric permittivity of water (ϵ≈80), $\epsilon_{0}$ is the dielectric permittivity of vacuum, $k_{B}$ is the Boltzmann constant, and *T* is the actual temperature. The electrostatic potential distribution Ψ(r) is used to describe the total electrostatic interactions in the system. The Ψ(r) can be found from the solution of the Poisson differential equation for spherical symmetry [87]

(10) $$\frac{d^{2}\psi \left(r\right)}{dr^{2}}+\frac{2}{r}\frac{d\psi (r)}{dr}=-kq\left(r\right)$$

Here, $\psi \left(r\right)=\;\left[e/k_{B}T\right]\Psi \left(r\right)$ is the dimensionless electrostatic potential, and $k=4\pi \lambda_{B}/dr$ is the dimensionless factor (dr [nm] is *r* increment). For the numerical solution of Equation (10), see our previous work [61]. The results for the electrostatic potential at T=310 K are shown in Figure 5c. For a model with the Gaussian smeared charge distribution over a soft bead [88] (from the center of this bead) the shape of the electrostatic potential is quite similar to the electrostatic potential in Figure 5c. This similarity is due to the fact, that the charge distribution q(r) (see Figure 5a) is very close to the form of the Gaussian charge distribution in the reciprocal space (see ref. [88]). The vertical dashed line indicates the position of the cumulative charge maximum corresponding to the position of the outer boundary of the dendrimer $R_{max}$ after which the diffusion layer begins. According to modern concepts, this region is a good approximation for the slip plane in which the zeta potential (ζ potential, see Table 4) is measured [89].

The dependence of the ratio $Q^{*}/Q_{bare}$ on temperature is shown in Figure 5d. The theoretically calculated points [90] are added for comparison. Both dendrimers have a similar temperature dependence of relative effective charge and the results obtained from simulation for Lys-2Arg (and Lys-2Lys) are in very good agreement with the theory.

**Figure 6:**
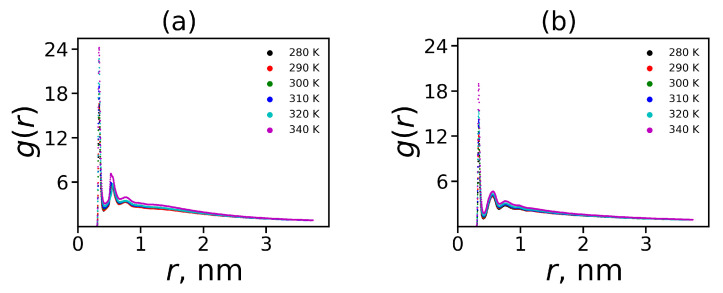
The ion pairs radial distribution function at different temperatures between (**a**) ions and the side charged groups; (**b**) ions and the terminal charged groups.

Electrostatic interactions lead to the formation of ion pairs between the positively charged dendrimer groups and the Cl-counterions. We calculated a pair correlation function g(r) for pairs between ions and charged side groups of spacers (Figure 6a) and the ions and charged terminal groups (Figure 6b). The first large peak of these functions corresponds to the formation of ion pairs. As can be seen from the Figure 6, the side groups form more ion pairs than the terminal groups. However, these results were obtained mainly due to the larger number of charged groups ($N_{ins}=28$) in 2Arg spacers than in Lys terminal groups ($N_{end}=16$).

**Figure 7:**
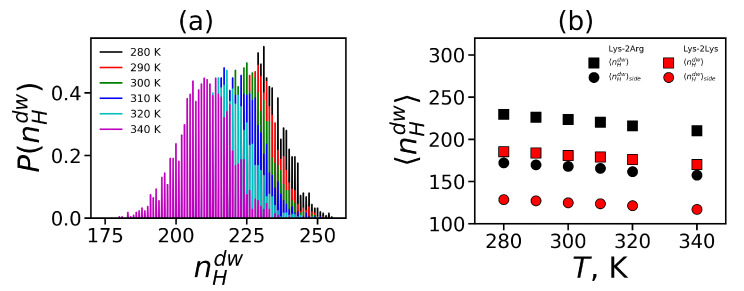
(**a**) The distribution of the dendrimer-water HB at different temperatures. (**b**) The average number of the dendrimer-water HB and the side segment-water HB as functions of temperature for the Lys-2Arg and Lys-2Lys dendrimers.

We have calculated the average number of ion pairs $\langle n_{ion\;pairs}\rangle$ by integrating the area under the first peak [61] in Figure 7. The counterions, which reduce the bare charge $Q_{bare}$ of the dendrimer to an effective charge $Q^{*}$, penetrate into the dendrimer and form two conditional groups: (i) ions in ion pairs, (ii) osmotic ions (these ions create osmotic pressure inside the dendrimer causing its swelling). The average number of the latter $\langle n_{ion\;osmotic}\rangle$ can be easily calculated by the equation $\langle n_{ion\;osmotic}\rangle =Q-Q^{*}-\langle n_{ion\;pairs}\rangle$. The average number of ion pairs $\langle n_{ion\;pairs}\rangle$ and the average number of osmotic ions $\langle n_{ion\;osmotic}\rangle$ inside dendrimers are presented in Table 4.

#### 3.2.3. The Hydrogen Bonds

In this subsection, we focus on calculating the number of hydrogen bonds (HB) that are intermediate in energy between strong and weak interactions. The formation of hydrogen bonds plays an important role in the stabilization of the secondary and tertiary structures of biomolecules [91,92]. The geometrical criteria [92,93] for hydrogen bond formation, which are commonly used in MD simulations, are as follows: the D-A distance must be less than 0.35 nm and the D-H-A angle must be less than 30${}^{\circ }$ [93].

First, we will consider the formation of hydrogen bonds between the atoms of the dendrimer and water. The distribution of these hydrogen bonds at different temperatures is shown in Figure 7a. It can be seen that with increasing temperature the distribution shifts toward a smaller number of hydrogen bonds.

In order to illustrate the effect of temperature on the formation of hydrogen bonds, we calculated the average number of all dendrimer-water HB $\langle n_{H}^{dw}\rangle$ and the side segment-water HB ${\langle n_{H}^{dw}\rangle }_{side}$ (see Figure 7b). Hydrogen bonds could exist also between donors and acceptors inside the dendrimer. We calculated the total average number of the intra-dendrimer HB $\langle n_{H}^{id}\rangle$ and the average number of hydrogen bonds between arginine residues ${\langle n_{H}^{id}\rangle }_{side}$ only. Among all the structural characteristics, only the number of hydrogen bonds decreases with increasing temperature, which is quite predictable. We do not observe a noticeable temperature dependence of the number of intramolecular HB in the dendrimer. Due to this reason, we present the average values of them in Table 5 only for T=310 K.

The hydrogen bonds maintain the stability of structures, therefore their lifetime [94,95,96] is an essential characteristic. There are different ways to estimate the HB lifetime [95,96]. In our analysis, we used the continuous hydrogen bond lifetimes [96]: the intra-dendrimer HB lifetime $\tau_{BF}^{id}$ and the dendrimer-water HB lifetime $\tau_{BF}^{dw}$. A sophisticated method for estimating these HB lifetimes is based on cubic spline interpolation described in [96]. The HB lifetimes obtained from the simulation data are presented in Table 5 for T=310 K. As we can see, the average lifetime of hydrogen bonds is the same in Lys-2Arg and Lys-2Lys.

### 3.3. The Mobility Characteristics Measured in NMR

**Figure 8:**
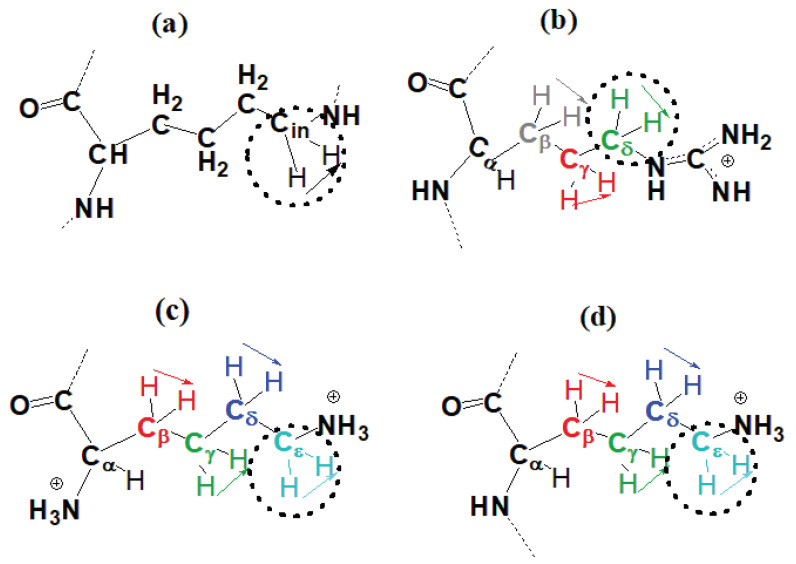
The structures of (**a**) inner Lys, (**b**) side Arg in spacers, and (**c**) terminal Lys segments of the Lys-2Arg dendrimer and (**d**) side Lys segment in spacers of the Lys-2Lys dendrimer. The side CH${}_{2}$ groups located at different distance from the ends of the side segments of Lys-2Arg (**b**) and Lys-2Lys (**d**) are marked by different colors: at the distance (contour length) equal to the length of one chemical bond from the end—by blue color, two bonds—by navy, three bonds—by green, four bonds—by red, five bonds—by gray.

In this subsection, we calculate the orientational mobility of the specific CH${}_{2}$ groups chemically connected to N atoms (marked by dashed circles in Figure 8). In NMR experiment the signals of CH2-N groups can be measured separately. These NMR active CH${}_{2}$ groups belong to three different types of aminoacid residues of Lys-2Arg dendrimer(inner Lys (branching points) (Figure 8a), side Arg (spacers) (Figure 8b) and terminal Lys (Figure 8c). The NMR data for these types of CH${}_{2}$ groups in the Lys-2Arg dendrimer were obtained previously [53].

**Figure 9:**
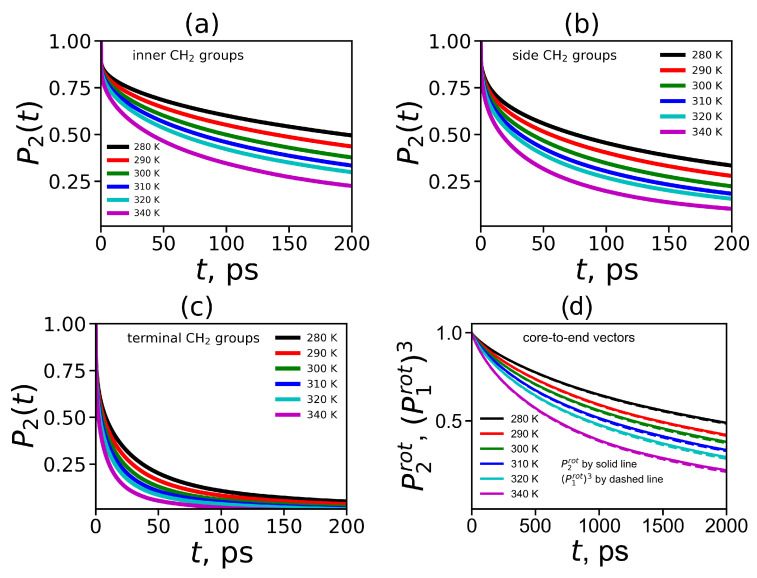
The time dependencies of ACF $P_{2}\left(t\right)$ for (**a**) inner, (**b**) side, (**c**) terminal CH${}_{2}$ groups. (**d**) The time dependencies of ACFs $P_{2}^{rot}\left(t\right)$ and ${\left(P_{1}^{rot}\left(t\right)\right)}^{3}$ for the core-to-end vectors. All data are presented for the Lys-2Arg dendrimer at the temperature range from 280 to 340 K.

To obtain the orientational mobility of the H-H vector in the CH${}_{2}$ groups (marked by dashed circles in Figure 8) from MD simulation, we calculated the second-order autocorrelation function ACF $P_{2}\left(t\right)$ (Equation (4)) for the H-H vectors in these groups by the same way as we did it for the Lys-2Lys dendrimer in [61]. The time dependencies of ACFs $P_{2}\left(t\right)$ for the H-H vectors in three different CH${}_{2}$ groups located at inner Lys (Figure 8a), side Arg (Figure 8b) and terminal Lys segments (Figure 8c) are shown in Figure 9a–c. Figure 9d demonstrates ACFs $P_{2}\left(t\right)$ for the core-to-end vectors. The time dependencies were calculated at different temperatures in the range from 280 K to 340 K.

It easy to see that $P_{2}\left(t\right)$ for all vectors decrease faster with increasing temperature. The $P_{2}\left(t\right)$ for the H-H vectors of the inner CH${}_{2}$ groups decrease very slowly in comparison to the same curves for the terminal CH${}_{2}$ groups. However, in the Lys-2Arg dendrimer the $P_{2}\left(t\right)$ for the H-H vectors of the side CH${}_{2}$ groups is close to the $P_{2}\left(t\right)$ for the inner CH${}_{2}$ groups. This fact is in contrast to the results for the Lys-2Lys dendrimer [52,61] where $P_{2}\left(t\right)$ functions for the side and terminal CH${}_{2}$ groups are practically the same. The $P_{2}\left(t\right)$ functions for the core-to-end vectors (Figure 9d) decay at least ten times slower than ones for the inner CH${}_{2}$ groups (Figure 9a). In addition to ACF $P_{2}\left(t\right)$, for the core-to-end vector, we calculated ACF $P_{1}\left(t\right)$ and $P_{1}^{3}\left(t\right)$. In several papers on the simulation of polymers and dendrimers [43,61,97] it was shown that there is a simple relationship between the 1st order ACF $P_{1}\left(t\right)$ and the 2nd order ACF $P_{2}\left(t\right)$:

(11) $$P_{2}\left(t\right)={\left(P_{1}\left(t\right)\right)}^{3}$$

As we can see from Figure 9d the time dependencies of $P_{1}^{3}\left(t\right)$ and $P_{2}\left(t\right)$ almost coincide at all temperatures. Thus the relationship (11) is valid for the core-to-end vector of the Lys-2Arg dendrimer as in other lysine based dendrimers studied earlier.

To compare the mobility characteristics $P_{2}\left(t\right)$ obtained from MD simulation and NMR experiments we calculated the reduced spin-lattice relaxation rate $\left[1/T_{1H}\right]$ in the susceptibility representation. This characteristic is a function of the variable ω (the angular frequency of NMR spectrometer) and is calculated using a linear combination of the spectral densities J(ω) and J(2ω) [98,99]:

(12) $$\left[\frac{1}{T_{1H}}\right]\left(\omega \right)=\omega \left(J\left(\omega \right)+4J\left(2\omega \right)\right)$$

The spectral density is calculated by the cosine Fourier transform

(13) $$J\left(\omega \right)=2\int_{0}^{\infty }P_{2}\left(t\right)\cos\left(\omega t\right)dt$$

**Figure 10:**
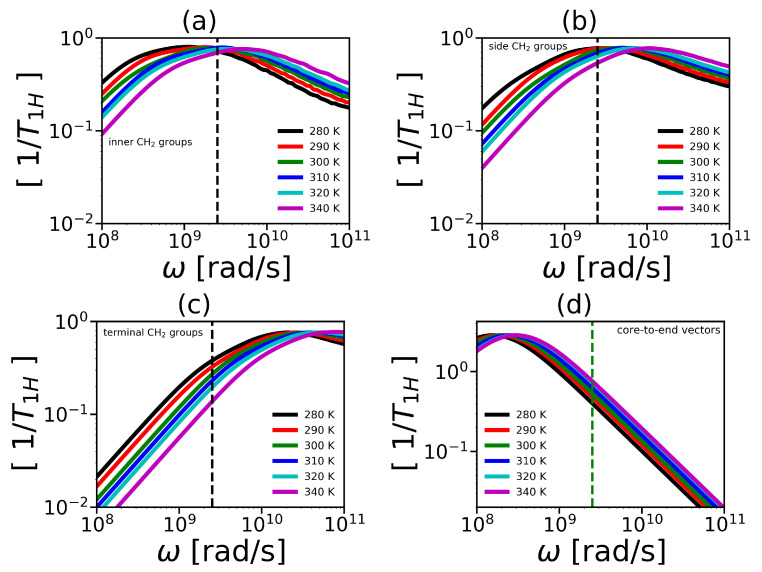
The frequency dependencies of $\left[1/T_{1H}\right]$ for (**a**) inner, (**b**) side, (**c**) terminal CH${}_{2}$ groups; and (**d**) core-to-end vector. The vertical line correspond to the frequency $\omega_{H}/2\pi =400$ MHz.

The frequency dependencies of $\left[1/T_{1H}\right]$ at different temperatures are presented in Figure 10. The vertical dashed line in these plots corresponds to the frequency ($\omega_{H}/2\pi =400$ MHz) of the spectrometer [52,53]. We can see from these plots that frequency dependencies of $\left[1/T_{1H}\right]$ change with temperature. Similarly to the time dependencies, these dependencies for the side groups of 2Arg spacers (Figure 10b) are very similar to $\left[1/T_{1H}\right]$ dependencies for the inner groups (Figure 10a) and differ significantly from those for the terminal ones (Figure 10c). At the same time, for the Lys-2Lys dendrimer studied earlier [52,61], the $\left[1/T_{1H}\right]$ dependencies for the side groups of 2Lys spacers were close to the $\left[1/T_{1H}\right]$ dependencies for the terminal groups. This large difference in the $\left[1/T_{1H}\right]$ behavior of these dendrimers obtained from our MD simulations is very strange. However, this is in good agreement with the NMR experimental results [52,53]. We will discuss this difference in detail later in this paper.

In the NMR experiment, the temperature dependence of $1/T_{1H}$ is measured at the given frequency of the spectrometer. Therefore, in order to obtain a similar dependence from the simulation, we calculate the position of $\left[1/T_{1H}\right]$ at the frequency of the spectrometer at each temperature. We converted the dimensionless of the reduced spin-lattice relaxation rate $\left[1/T_{1H}\right]$ to the susceptibility representation of the $1/T_{1H}$ (measured in the NMR experiment) using the following relationship:

(14) $$\frac{1}{T_{1H}}=\frac{A_{0}}{\omega_{H}}\left[\frac{1}{T_{1H}}\right]$$

where $A_{0}$ is the constant determined by the quantum chemistry parameters and does not depend on frequency or temperature. The theoretical value of $A_{0}$ for CH${}_{2}$ groups $A_{0}^{theory}$ is equal to $0.56\times 10^{10}$ s${}^{-2}$. At the same time, $A_{0}$ is often used as a fitting parameter (see for example [43,61]). For calculation of the temperature dependence of $1/T_{1H}$ we used the theoretical value $A_{0}^{theory}$ for all CH${}_{2}$ groups except the terminal CH${}_{2}$ groups of Lys-2Arg (for which the value $A_{0}=0.88\cdot 10^{10}$ s${}^{-2}$ was used).

**Figure 11:**
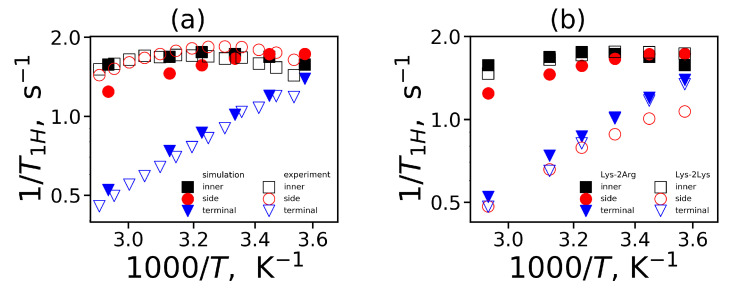
The ${}^{1}$H NMR spin-lattice relaxation rate $1/T_{1H}$ as a function of inverse temperature 1000/T for the inner, side and terminal CH${}_{2}$ groups at the fixed frequency $\omega_{H}/2\pi =400$ MHz: (**a**) from the simulation and experimental data for Lys-2Arg, (**b**) from the simulation data for Lys-2Arg and Lys-2Lys.

The temperature dependencies of $1/T_{1H}$ for the CH${}_{2}$ groups in the Lys-2Arg and Lys-2Lys dendrimers are plotted in Figure 11. The new simulation results and the experimental NMR data for three types of the CH${}_{2}$ groups (inner, side and terminal) in the Lys-2Arg dendrimer [53] are in very good agreement Figure 11a. The temperature dependencies of $1/T_{1H}$ for the side CH${}_{2}$ groups in the Lys-2Arg dendrimer differ from those for the terminal CH${}_{2}$ groups. At the same time in our previous NMR and simulation papers [52,61], for the Lys-2Lys dendrimer the temperature dependencies of $1/T_{1H}$ for the side and terminal CH${}_{2}$ groups were practically the same. However, in MD simulation we can calculate the mobility of the terminal and side groups of the Lys-2Lys dendrimer separately. We compared the simulation data both for the Lys-2Arg and Lys-2Lys dendrimers in Figure 11b. We can conclude that the temperature dependency of $1/T_{1H}$ of the side CH${}_{2}$ groups in 2Lys spacers of the Lys-2Lys dendrimer is close to those of the terminal CH${}_{2}$ groups of both dendrimers. However, the mobility of the side CH${}_{2}$ groups of 2Arg spacers of the Lys-2Arg dendrimer is close to the mobility of the inner CH${}_{2}$ groups of both dendrimers. Thus, the difference between the mobility of the side CH${}_{2}$ groups in 2Arg and 2Lys spacers is not only quantitative but also qualitative. To understand the reason of this difference we performed the calculation of the mobility characteristics for all CH${}_{2}$ groups in spacers.

### 3.4. The Mobility Characteristics of All CH${}_{2}$ Groups in Side Segments of Spacers from MD Simulation

From previous papers on NMR and MD simulation of different lysine dendrimer [43,61], it was established that the mobility of the inner CH${}_{2}$ groups significantly differs from the mobility of the terminal groups in the same dendrimers. In [53] and in the present paper we have obtained a similar result for the Lys-2Arg dendrimer both from the NMR experiment and from the simulation. The mobility of the CH${}_{2}$ groups of the Lys-2Lys dendrimers was practically the same as the mobility of the terminal groups [52,61]. However, in the Lys-2Arg dendrimer both the NMR experiment [53] and the MD simulation demonstrate that the mobility of the side CH${}_{2}$ groups significantly differ from the mobility of the terminal CH${}_{2}$ groups and is close to the mobility of the inner CH${}_{2}$ groups.

It was assumed [53] that a possible reason for this difference in the mobility of the side segments in the Lys-2Arg and Lys-2Lys dendrimers [52,53] could be the arginine-arginine pairing [57,58], which leads to cross-linking of the Lys-2Arg dendrimer branches. The Arg-Arg pairing effect is well known for arginine dimers and short linear arginine homopeptides. However, it is not clear whether it can play a significant role in the case of dendrimers or not. Another possible reason is that the NMR active CH${}_{2}$ groups (C${}_{\delta }$H${}_{2}$ in Figure 8b) in 2Arg spacers of Lys-2Arg and CH${}_{2}$ groups in 2Lys spacers (C${}_{\epsilon }$H${}_{2}$ in Figure 8c) in Lys-2Lys occupy the different structural positions from the ends of the side segments (the topological distance). In other words, the topological distance from the C${}_{\delta }$H${}_{2}$ group to the end of the side Arg segment (three bonds) is not the same as the distance from the C${}_{\epsilon }$H${}_{2}$ group to the end of side Lys segment (two bonds).

If the difference between the mobility of the side segments of Lys-2Arg and Lys-2Lys is due to the Arg-Arg pairing or other types of interaction that lead to effective cross-linking of the neighboring branches of Lys-2Arg, then the orientational mobility of the different CH${}_{2}$ groups of the side segments will be approximately the same. Otherwise, we can observe the dependence of the mobility of the different CH${}_{2}$ groups in the same side segment (Arg or Lys) on the distance from a particular CH${}_{2}$ group to the end of this side segment.

To understand the reason for the different mobility of the NMR active CH${}_{2}$ groups, from the MD simulation we calculated the mobility of all CH${}_{2}$ groups in each Arg (C${}_{\beta }$H${}_{2}$, C${}_{\gamma }$H${}_{2}$, C${}_{\delta }$H${}_{2}$, Figure 8b) and Lys segment (C${}_{\beta }$H${}_{2}$, C${}_{\gamma }$H${}_{2}$, C${}_{\delta }$H${}_{2}$, C${}_{\epsilon }$H${}_{2}$, Figure 8d) of spacers.

**Figure 12:**
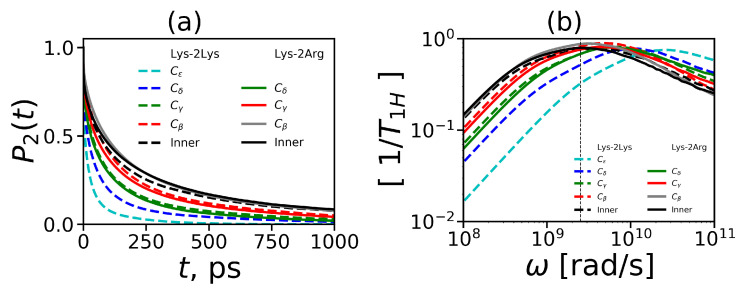
(**a**) The 2nd order ACF $P_{2}\left(t\right)$ and (**b**) the frequency dependencies of $\left[1/T_{1H}\right]$ in the susceptibility representation for H-H vector in the inner and the different types of CH${}_{2}$ groups in side segments at T=310 K.

To characterize the mobility of these additional CH${}_{2}$ groups we calculated the 2nd order ACF $P_{2}\left(t\right)$. The results of these calculations are presented in Figure 12a. It can be seen that the time dependencies of $P_{2}\left(t\right)$ for the CH${}_{2}$ groups located at different topological distances from the end of the side segment in the Lys-2Arg (solid lines) and Lys-2Lys (dashed lines) dendrimers differ. It is important to note that the $P_{2}\left(t\right)$ for the CH${}_{2}$ groups of these dendrimers located at the same topological distance (marked by the same color in Figure 12a and in Figure 8) practically coincide. The first of these two results means that our suggestion about the possible cross-linking of dendrimer branches due to the arginine-arginine pairing is not valid (otherwise, $P_{2}\left(t\right)$ for all CH${}_{2}$ groups in spacers of the same dendrimer should be approximatly the same).

The second result means that the topological distance is the main parameter which determines the NMR relaxation of the H-H vectors in CH${}_{2}$ groups of both spacers.

For illustration of these results, we performed a rough evaluation of the relaxation times for each CH${}_{2}$ group in 2Arg and 2Lys spacers of the dendrimers under study. In Table 6, the results of this evaluation are presented as a function of the number of chemical bonds from the C atom of a given CH${}_{2}$ group to the N atom of the end NH${}_{2}$ (for Arg) or the end NH${}_{3}$ group (for Lys) in the side segments of spacers in the Lys-2Arg or Lys-2Lys dendrimers, correspondingly. In the side segments of 2Arg spacers, there are the CH${}_{2}$ groups located at the topological distances equal to the length of 3, 4 and 5 chemical bonds. In the side segments of 2Lys spacers, there are the CH${}_{2}$ groups located at the topological distances equal to the length of 1, 2, 3, and 4 chemical bonds. The shortest and longest (the statistical error is about 10%) characteristic times for the terminal and inner CH${}_{2}$ groups in each dendrimer are presented in the second and last columns in Table 6, respectively. It is easy to see that characteristic times arranged in this way are very similar for both dendrimers.

In NMR experiments [52,53] we considered the side CH2 groups that remoted from the end of the side segment at the distance of one (for Lys-2Lys) and three (for Lys-2Arg) chemical bonds. Therefore, the reason for the difference in the spin-lattice relaxation rates of these side groups measured by NMR [52,53] is the different topological distance.

To compare our simulation results and the experimental NMR data for all CH${}_{2}$ groups in side segments of 2Arg and 2Lys spacers of both dendrimers, we converted the calculated time dependencies of $P_{2}\left(t\right)$ to the frequency dependencies of $\left[1/T_{1H}\right]$ (using Equation (12)). The frequency dependencies of $\left[1/T_{1H}\right]$ for these CH${}_{2}$ groups are shown in Figure 12b. We can see that the frequency dependencies of $\left[1/T_{1H}\right]$ for the CH${}_{2}$ groups with the different structural positions differ within the same dendrimer. However, the frequency dependencies of $\left[1/T_{1H}\right]$ for the CH${}_{2}$ groups located at the same topological distance from the ends of the side segments in these two dendrimers practically coincide. This result confirms our conclusion, based on the comparison of the time dependencies of $P_{2}\left(t\right)$ for the NMR active CH${}_{2}$ groups in 2Arg and 2Lys spacers, that the difference in the mobility of these groups is due to their different topological positions from the end of the corresponding side segment.

**Figure 13:**
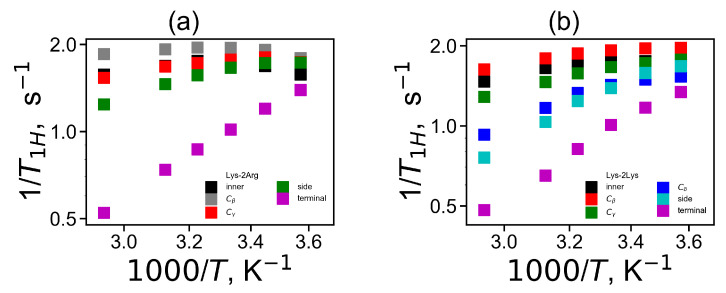
The ${}^{1}$H NMR spin-lattice relaxation rate $1/T_{1H}$ as a function of inverse temperature 1000/T for the different CH${}_{2}$ groups at the fixed frequency $\omega_{H}/2\pi =400$ MHz for (**a**) Lys-2Arg, (**b**) Lys-2Lys.

Finally, we calculated the temperature dependencies of the spin-lattice NMR relaxation rate $1/T_{1H}$ for all side CH${}_{2}$ groups in 2Arg and 2Lys spacers of both dendrimers by analogy with [52,53]. These dependencies are presented in Figure 13a for the Lys-2Arg dendrimer and in Figure 13b for the Lys-2Lys dendrimer. According to these figures, in general, the temperature dependencies of $1/T_{1H}$ looks rather similar for both dendrimers if we compare, for instance, the curves for the side CH${}_{2}$-N groups.

We see that with distance from the end of the side segment, the mobility decreases. This behavior is related to the NMR semiflexibility effect of hyperbranched macromolecules [100,101,102] and is discussed in some detail for lysine dendrimers [43].

## 4. Conclusions

Recently, it was shown that a novel peptide dendrimer with Lys-2Arg repeating units developed for gene delivery has better transport properties than the new similar dendrimers with Lys-2Gly and Lys-2Lys repeating units [55,56]. In this work, we performed MD simulation of the Lys-2Arg dendrimer studied earlier by NMR [53]. Moreover, we compared the structure and the mobility of Lys-2Arg with characteristics of the Lys-2Lys dendrimer obtained from the previous NMR experiments and MD simulation [52,61]. We found that the size and shape of the Lys-2Arg dendrimer are very close to those of Lys-2Lys. The internal structure of both dendrimers is similar. The terminal groups of these dendrimers are evenly distributed over the surface of both dendrimers. Lys-2Arg and Lys-2Lys have similar electrostatic characteristics (charge distribution, zeta potential etc.). Most of the structural and electrostatic properties of both dendrimers are also independent of temperature, i.e., these dendrimers form similar stable nanocontainers.

The local orientational mobility of the inner and terminal lysine groups in each dendrimer is different. In addition, the difference in the mobility is approximately the same in both dendrimers and very close to that one obtained from NMR [52,53]. However, the MD simulation carried out in this paper confirms the significant difference in the mobility of the side CH${}_{2}$-N groups in the Lys-2Arg dendrimer in comparison with the same CH${}_{2}$-N groups in the Lys-2Lys dendrimer [52,53]. We have revealed that this difference is due to the larger distance from the NMR active side CH${}_{2}$-N group to the end of the side segment in the 2Arg spacer than the distance from a similar group in 2Lys spacer. We obtained that the Lys-2Arg and Lys-2Lys dendrimers are difficult to distinguish by measuring their properties. Nevertheless, the difference in the mobility of the side groups CH${}_{2}$-N in spacers of these similar dendrimers makes it possible to detect and distinguish them in aqueous solution by NMR.

## Figures and Tables

**Table 1:** The characteristics of Lys-2Arg dendrimer: the molecular mass *M*, the charge $Q_{bare}$, the number of terminal NH${}_{3}^{+}$ groups $N_{end}$ and their charge $Q_{end}$, also the number $N_{ins}$ and the charge $Q_{ins}$ of inserted aminoacid residues, and the total number $N_{H_{2}O}$ of water molecules in the system.

Dendrimer	*M* (g/mol)	$Q_{bare}$ (e)	$N_{end}$	$Q_{end}$ (e)	$N_{ins}$	$Q_{ins}$ (e)	$N_{H_{2}O}$
Lys-2Arg	6479.64	+44	16	+16	28	+28	13,182

**Table 2:** The characteristic times $\tau_{rot}^{P_{1}}$ (ns) of the Lys-2Arg and Lys-2Lys dendrimers.

Temperature	Lys-2Arg	Lys-2Lys
280 K	11.9	13.7
290 K	10.0	10.5
300 K	8.9	9.0
310 K	7.5	7.0
320 K	6.4	5.5
340 K	6.1	5.1

**Table 3:** The characteristics of the Lys-2Arg and Lys-2Lys dendrimers: the asphericity parameter α, the radius of inertia $R_{g}$ (nm), the hydrodynamic radius $R_{h}$ (nm), the ratio $R_{h}/R_{g}$, the rigid-sphere-approximation $\sqrt{5/3}R_{g}$ (nm), the position of terminal groups $R_{e}$ (nm), the radius $R_{max}$ (nm) and the congretation coefficient $k_{45}$ at temperature 310 K.

Dendrimer	α	$R_{g}$	$R_{h}$	$R_{h}/R_{g}$	$\sqrt{5/3}R_{g}$	$R_{e}$	$R_{\max}$	$k_{45}$
Lys-2Arg	0.02	2.0	1.6	0.8	2.6	2.6	3.1	0.1
Lys-2Lys	0.02	2.0	1.7	0.8	2.6	2.7	3.1	0.1

**Table 4:** The local characteristics of Lys-2Arg and Lys-2Lys dendrimers: the average number of ion pairs $\langle n_{ion\;pairs}\rangle$ between the counterions and the charged groups in the dendrimer, the effective dendrimer charge $Q^{*}$ (e), the degree of a charge renormalization of the dendrimer $Q^{*}/Q_{bare}$, the surface charge density σ (e/nm${}^{2}$), and ζ potential (mV) at temperature T=310 K.

Dendrimer	$Q^{*}$	$Q^{*}/Q_{\mathrm{bare}}$	σ	ζ	$\langle n_{\mathrm{ion}\;\mathrm{pairs}}\rangle$	$\langle n_{\mathrm{ion}\;\mathrm{osmotic}}\rangle$
Lys-2Arg	13.7	0.3	0.1	17.4	6.2	24.1
Lys-2Lys	13.2	0.3	0.1	17.3	5.7	25.1

**Table 5:** The hydrogen bond characteristics of Lys-2Arg and Lys-2Lys: the average number of the inter-dendrimer HB $\langle n_{H}^{id}\rangle$ and the dendrimer-water HB $\langle n_{H}^{dw}\rangle$, HB between the side segments and water ${\langle n_{H}^{dw}\rangle }_{side}$ (per side segment ${\langle n_{H}^{dw}\rangle }_{side}/N_{ins}$); the lifetime of the intra-dendrimer HB $\tau_{BF}^{id}$ and the dendrimer-water HB $\tau_{BF}^{dw}$ at T=310 K.

Dendrimer	$\langle n_{H}^{\mathrm{id}}\rangle$	$\langle n_{H}^{\mathrm{dw}}\rangle$	${\langle n_{H}^{\mathrm{dw}}\rangle }_{\mathrm{side}}$	${\langle n_{H}^{\mathrm{dw}}\rangle }_{\mathrm{side}}/N_{\mathrm{ins}}$	$\tau_{\mathrm{BF}}^{\mathrm{id}}$ (ps)	$\tau_{\mathrm{BF}}^{\mathrm{dw}}$ (ps)
Lys-2Arg	3.7	220.5	165.9	5.9	110	51
Lys-2Lys	1.5	179.2	123.9	4.4	110	51

**Table 6:** The characteristic times [ps] of different CH${}_{2}$ groups in the side segments of the Lys-2Arg and Lys-2Lys dendrimers at *T* = 310 K.

Dendrimer	Terminal	1 Bond	2 Bonds	3 Bonds	4 Bonds	5 Bonds	Inner
Lys-2Arg	10	-	-	70	100	165	160
Lys-2Lys	10	15	20	70	110	-	140

## References

[1] Buhleier E., Wehner W., Vogtle F. (1978). Cascade and Nonskid-Chain-Like Syntheses of Molecular Cavity Topologies. Synthesis.

[2] Tomalia D., Baker H., Dewald M., Hall J., Kallos G., Martin S., Roeck J., Ryder J., Smit P. (1985). A New Class of Polymers: Starburst-Dendritic Macromolecules. Polym. J..

[3] Hawker C.J., Frechet J.M.J. (1990). Control of surface functionality in the synthesis of dendritic macromolecules using the convergent-growth approach. Macromolecules.

[4] Patel H.N., Patel P.M. (2013). Dendrimer applications—A review. Int. J. Pharm. Bio Sci..

[5] Abbasi E., Aval S., Akbarzadeh A., Milani M., Nasrabadi H., Joo S., Hanifehpour Y., Nejati-Koshki K., Pashaei-Asl R. (2014). Dendrimers: Synthesis, applications, and properties. Nanoscale Res. Lett..

[6] Lyu Z., Ding L., Huang A.T., Kao C.L., Peng L. (2019). Poly(amidoamine) dendrimers: Covalent and supramolecular synthesis. Mater. Today Chem..

[7] Sadler K., Tam J.P. (2002). Peptide dendrimers: Applications and synthesis. Rev. Mol. Biotechnol..

[8] Yemul O., Imae T. (2008). Synthesis and characterization of poly(ethyleneimine) dendrimers. Colloid Polym. Sci..

[9] Schlenk C., Frey H. (1999). Carbosilane Dendrimers—Synthesis, Functionalization, Application. Silicon Chemistry.

[10] Svenson S. (2009). Dendrimers as versatile platform in drug delivery applications. Eur. J. Pharm. Biopharm..

[11] Rabiee N., Ahmadvand S., Ahmadi S., Fatahi Y., Dinarvand R., Bagherzadeh M., Hamblin M.R. (2020). Carbosilane Dendrimers: Drug and Gene Delivery Applications. J. Drug Deliv. Sci. Technol..

[12] Cavazzana-Calvo M., Thrasher A., Mavilio F. (2004). The future of gene therapy. Nature.

[13] Davis M., Zuckerman J., Choi C., Seligson D., Tolcher A., Alabi C., Yen Y., Heidel J., Ribas A. (2010). Evidence of RNAi in humans from systemically administered siRNA via targeted nanoparticles. Nature.

[14] Stone D. (2010). Novel viral vector systems for gene therapy. Viruses.

[15] Yin H., Kanasty R.L., Eltoukhy A.A., Vegas A.J., Dorkin J.R., Anderson D.G. (2014). Non-viral vectors for gene-based therapy. Nat. Rev. Genet..

[16] Jiang D., Wang M., Wang T., Zhang B., Liu C., Zhang N. (2017). Multifunctionalized polyethyleneimine-based nanocarriers for gene and chemotherapeutic drug combination therapy through one-step assembly strategy. Int. J. Nanomed..

[17] Kuboyama T., Yagi K., Naoi T., Era T., Yagi N., Nakasato Y., Yabuuchi H., Takahashi S., Shinohara F., Iwai H. (2019). Simplifying the Chemical Structure of Cationic Lipids for siRNA Lipid Nanoparticles. ACS Med. Chem. Lett..

[18] Zhang X., Duan Y., Wang D., Bian F. (2015). Preparation of arginine modified PEI-conjugated chitosan copolymer for DNA delivery. Carbohydr. Polym..

[19] Lehto T., Ezzat K., Wood M.J.A., El Andaloussi S. (2016). Peptides for nucleic acid delivery. Adv. Drug Deliv. Rev..

[20] Li F., Li Y., Zhou Z., Lv S., Deng Q., Xu X., Yin L. (2017). Engineering the Aromaticity of Cationic Helical Polypeptides toward “Self-Activated” DNA/siRNA Delivery. ACS Appl. Mater. Interfaces.

[21] Martinez-Fong D., Mullersman J.E., Purchio A.F., Armendariz-Borunda J., Martinez-Hernandez A. (1994). Non-enzymatic glycosylation of poly-L-lysine: A new tool for targeted gene delivery. Hepatology.

[22] Zhang X., Oulad-Abdelghani M., Zelkin A.N., Wang Y., Haîkel Y., Mainard D., Voegel J.C., Caruso F., Benkirane-Jessel N. (2010). Poly(L-lysine) nanostructured particles for gene delivery and hormone stimulation. Biomaterials.

[23] Bikram M., Cheol-Hee A.C.H., Chae S.Y., Lee M., Yockman J.W., Kim S.W. (2004). Biodegradable poly(ethylene glycol)-co-poly(l-lysine)-g-histidine multi-block copolymers for non-viral gene delivery. Macromolecules.

[24] Dufes C., Uchegbu I., Schatzlein A.G. (2005). Dendrimers in gene delivery. Adv. Drug Deliv. Rev..

[25] Zhou J., Wu J., Hafdi N., Behr J.P., Erbacher P., Peng L. (2006). PAMAM dendrimers for efficient siRNA delivery and potent gene silencing. Chem. Commun..

[26] Biswas S., Torchilin V. (2013). Dendrimers for siRNA delivery. Pharmaceuticals.

[27] Sheikhi Mehrabadi F., Zeng H., Johnson M., Schlesener C., Guan Z., Haag R. (2015). Multivalent dendritic polyglycerolamine with arginine and histidine end groups for efficient siRNA transfection. Beilstein J. Org. Chem..

[28] Lee H., Choi J.S., Larson R.G. (2011). Molecular Dynamics Studies of the Size and Internal Structure of the PAMAM Dendrimer Grafted with Arginine and Histidine. Macromolecules.

[29] Denkewalter R.G., Kolc J., Lukasavage W.J. (1981). Macromolecular Highly Branched Homogeneous Compound Based on Lysine Units. U.S. Patent.

[30] Ohsaki M., Okuda T., Wada A., Hirayama T., Niidome T., Aoyagi H. (2002). In vitro gene transfection using dendritic poly (L-lysine). Bioconjug. Chem..

[31] Vlasov G., Korol’kov V., Pankova G., Tarasenko I., Baranov A., Glazkov P., Kiselev A., Ostapenko O., Lesin E., Baranov V. (2004). Lysine Dendrimers and Their Starburst Polymer Derivatives: Possible Application for DNA Compaction and in vitro Delivery of Genetic Constructs. Russ. J. Bioorg. Chem..

[32] Neuhaus B., Tosun B., Rotan O., Frede A., Westendorf A.M., Epple M. (2016). Nanoparticles as transfection agents: A comprehensive study with ten different cell lines. RSC Adv..

[33] Rewatkar P.V., Sester D.P., Parekh H.S., Parat M.O. (2016). Express in Vitro Plasmid Transfection Achieved with 16+ Asymmetric Peptide Dendrimers. ACS Biomater. Sci. Eng..

[34] Wang S., Chen R. (2017). PH-responsive, lysine-based, hyperbranched polymers mimicking endosomolytic cell-penetrating peptides for efficient intracellular delivery. Chem. Mater..

[35] Vlasov G., Pankova G., Nikonova I., Antonov N.G. (2003). Starburst Conjugates of Proteins with Carbon-Chain Polymers Containing Low Molecular Biologically Active Compounds: Synthesis and Immunogenicity. Russ. J. Bioorg. Chem..

[36] Okuda T., Sugiyama A., Niidome T., Aoyagi H. (2004). Characters of dendritic poly(L-lysine) analogues with the terminal lysines replaced with arginines and histidines as gene carriers in vitro. Biomaterials.

[37] Luo K., Li C., Li L., She W., Wang G., Gu Z. (2012). Arginine functionalized peptide dendrimers as potential gene delivery vehicles. Biomaterials.

[38] Kozhikhova K.V., Andreev S.M., Shilovskiy I.P., Timofeeva A.V., Gaisina A.R., Shatilov A.A., Turetskiy E.A., Andreev I.M., Smirnov V.V., Dvornikov A.S. (2018). A novel peptide dendrimer LTP efficiently facilitates transfection of mammalian cells. Org. Biomol. Chem..

[39] Tam J. (1988). Synthetic peptide vaccine design: Synthesis and properties of a high-density multiple antigenic peptide system. Proc. Natl. Acad. Sci. USA.

[40] Aharoni S.M., Crosby C.R., Walsh E.K. (1982). Size and solution properties of globular tert-butyloxycarbonyl-poly(*α*,*ϵ*-L-lysine). Macromolecules.

[41] Aharoni S., Murthy M. (1983). Spherical non-draining BOC-poly-(*α*,*ϵ*-L-lysine) macromolecules SAX and viscous study. Polym. Commun..

[42] Vlasov G., Pavlov G., Bayanova N., Korneeva E., Ebel C., Khodorkovskii M., Artamonova T. (2004). Dendrimers Based on a-Amino Acids: Synthesis and Hydrodynamic Characteristics. Dokl. Phys. Chem..

[43] Markelov D.A., Falkovich S.G., Neelov I.M., Ilyash M.Y., Matveev V.V., Lahderanta E., Ingman P., Darinskii A.A. (2015). Molecular dynamics simulation of spin–lattice NMR relaxation in poly-l-lysine dendrimers: Manifestation of the semiflexibility effect. Phys. Chem. Chem. Phys..

[44] Roberts B.P., Scanlon M.J., Krippner G.Y., Chalmers D.K. (2009). Molecular Dynamics of Poly(l-lysine) Dendrimers with Naphthalene Disulfonate Caps. Macromolecules.

[45] Neelov I., Falkovich S., Markelov D., Paci E., Darinskii A., Tenhu H. (2013). Molecular Dynamics of Lysine Dendrimers. Computer Simulation and NMR. Dendrimers in Biomedical Applications.

[46] Neelov I., Markelov D., Falkovich S., Ilyash M., Okrugin B., Darinskii A. (2013). Mathematical simulation of lysine dendrimers. Temperature dependencies. Polym. Sci. Ser. C.

[47] Falkovich S., Markelov D., Neelov I., Darinskii A. (2013). Are structural properties of dendrimers sensitive to the symmetry of branching? Computer simulation of lysine dendrimers. J. Chem. Phys..

[48] Maillard N., Clouet A., Darbre T., Reymond J.L. (2009). Combinatorial libraries of peptide dendrimers: Design, synthesis, on-bead high-throughput screening, bead decoding and characterization. Nat. Protoc..

[49] Luo K., Li C., Wang G., Nie Y., He B., Wu Y., Gu Z. (2011). Peptide dendrimers as efficient and biocompatible gene delivery vectors: Synthesis and in vitro characterization. J. Control. Release.

[50] Kwok A., Eggimann G.A., Reymond J.L., Darbre T., Hollfelder F. (2013). Peptide dendrimer/lipid hybrid systems are efficient DNA transfection reagents: Structure–activity relationships highlight the role of charge distribution across dendrimer generations. ACS Nano.

[51] Santos S., Gonzaga R., Silva J., Savino D., Prieto D., Shikay J., Silva R., Paulo L., Ferreira E., Giarolla J. (2017). Peptide dendrimers: Drug/gene delivery and other approaches. Can. J. Chem..

[52] Sheveleva N.N., Markelov D.A., Vovk M.A., Mikhailova M.E., Tarasenko I.I., Neelov I.M., Lahderanta E. (2018). NMR studies of excluded volume interactions in peptide dendrimers. Sci. Rep..

[53] Sheveleva N.N., Markelov D.A., Vovk M.A., Mikhailova M.E., Tarasenko I.I., Tolstoy P.M., Neelov I.M., Lähderanta E. (2019). Lysine-based dendrimer with double arginine residues. RSC Adv..

[54] Sheveleva N.N., Markelov D.A., Vovk M.A., Tarasenko I.I., Mikhailova M.E., Ilyash M.Y., Neelov I.M., Lahderanta E. (2019). Stable Deuterium Labeling of Histidine-Rich Lysine-Based Dendrimers. Molecules.

[55] Gorzkiewicz M., Konopka M., Janaszewska A., Tarasenko I.I., Sheveleva N.N., Gajek A., Neelov I.M., Klajnert-Maculewicz B. (2020). Application of new lysine-based peptide dendrimers D3K2 and D3G2 for gene delivery: Specific cytotoxicity to cancer cells and transfection in vitro. Bioorg. Chem..

[56] Gorzkiewicz M., Kopec O., Janaszewska A., Konopka M., Pedziwiatr-Werbicka E., Tarasenko I.I., Bezrodnyi V.V., Neelov I.M., Klajnert-Maculewicz B. (2020). Poly(lysine) Dendrimers Form Complexes with siRNA and Provide Its Efficient Uptake by Myeloid Cells: Model Studies for Therapeutic Nucleic Acid Delivery. Int. J. Mol. Sci..

[57] Vondrášek J., Mason P.E., Heyda J., Collins K.D., Jungwirth P. (2009). The Molecular Origin of Like-Charge Arginine-Arginine Pairing in Water. J. Phys. Chem. B.

[58] Lee D., Lee J., Seok C. (2013). What stabilizes close arginine pairing in proteins?. Phys. Chem. Chem. Phys..

[59] Filipe L.C.S., Machuqueiro M., Darbre T., Baptista A.M. (2016). Exploring the Structural Properties of Positively Charged Peptide Dendrimers. J. Phys. Chem. B.

[60] Heitz M., Zamolo S., Javor S., Reymond J.L. (2020). Fluorescent Peptide Dendrimers for siRNA Transfection: Tracking pH Responsive Aggregation, siRNA Binding and Cell Penetration. Bioconjug. Chem..

[61] Mikhtaniuk S.E., Bezrodnyi V.V., Shavykin O.V., Neelov I.M., Sheveleva N.N., Penkova A.V., Markelov D.A. (2020). Comparison of Structure and Local Dynamics of Two Peptide Dendrimers with the Same Backbone but with Different Side Groups in Their Spacers. Polymers.

[62] Mikhailov I., Darinskii A. (2014). Does symmetry of branching affect the properties of dendrimers?. Polym. Sci. Ser. A.

[63] Shavykin O., Neelov I., Darinskii A. (2016). Is the Manifestation of the Local Dynamics in the Spin-Lattice NMR Relaxation in Dendrimers Sensitive to Excluded Volume Interactions. Phys. Chem. Chem. Phys..

[64] Shavykin O., Mikhailov I., Darinskii A., Neelov I., Leermakers F. (2018). Effect of an asymmetry of branching on structural characteristics of dendrimers revealed by Brownian dynamics simulations. Polymer.

[65] Okrugin B., Neelov I., Leermakers F.M., Borisov O. (2017). Structure of asymmetrical peptide dendrimers: Insights given by self-consistent field theory. Polymer.

[66] Shavykin O.V., Leermakers F.A., Neelov I.M., Darinskii A.A. (2018). Self-Assembly of Lysine-Based Dendritic Surfactants Modeled by the Self-Consistent Field Approach. Langmuir.

[67] Shavykin O., Neelov I., Borisov O., Darinskii A., Leermakers F. (2020). SCF Theory of Uniformly Charged Dendrimers: Impact of Asymmetry of Branching, Generation Number, and Salt Concentration. Macromolecules.

[68] Abraham M.J., Murtola T., Schulz R., Pall S., Smith J.C., Hess B., Lindahl E. (2015). GROMACS: High performance molecular simulations through multi-level parallelism from laptops to supercomputers. Software X.

[69] Lindorff-Larsen K., Piana S., Palmo K., Maragakis P., Klepeis J.L., Dror R.O., Shaw D.E. (2010). Improved side-chain torsion potentials for the Amber ff99SB protein force field. Proteins.

[70] Neelov I.M., Binder K. (1995). Brownian dynamics of grafted polymer chains: Time-dependent properties. Macromol. Theory Simul..

[71] Neelov I., Adolf D., McLeish T., Paci E. (2006). Molecular dynamics simulation of dextran extension by constant force in single molecule AFM. Biophys. J..

[72] Neelov I., Adolf D. (2003). Brownian dynamics simulations of dendrimers under elongational flow: Bead-rod model with hydrodynamic interactions. Macromolecules.

[73] Neelov I., Adolf D. (2004). Brownian dynamics simulation of hyperbranched polymers under elongational flow. Phys. Chem. B.

[74] Gowdy J., Batchelor M., Neelov I., Paci E. (2017). Nonexponential kinetics of loop formation in proteins and peptides: A signature of rugged free energy landscapes?. J. Phys. Chem. B.

[75] Evans D., Holian B.L. (1985). The Nose–Hoover thermostat. J. Chem. Phys..

[76] Parrinello M., Rahman A. (1982). Polymorphic transitions in single crystals: A new molecular dynamics method. J. Appl. Phys..

[77] Kell G.S. (1970). Isothermal Compressibility of liquid Water at 1 Atm. J. Chem. Eng. Data.

[78] Narain R. (2016). Polymers and Nanomaterials for Gene Therapy.

[79] Maiti P., Cagin T., Wang G., Goddard W. (2004). Structure of PAMAM Dendrimers: Generations 1 through 11. Macromolecules.

[80] Klos J., Sommer J. (2009). Properties of Dendrimers with Flexible Spacer-Chains: A Monte Carlo Study. Macromolecules.

[81] Zacharopoulos N., Economou I. (2002). Morphology and Organization of Poly(propylene imine) Dendrimers in the Melt from Molecular Dynamics Simulation. Macromolecules.

[82] Theodorou D.N., Suter U.W. (1985). Shape of unperturbed linear polymers: Polypropylene. Macromolecules.

[83] Rudnick G., Gaspari G. (1986). The aspherity of random walks. J. Phys. A.

[84] Nygaard M., Kragelund B.B., Papaleo E., Lindorff-Larsen K. (2017). An Efficient Method for Estimating the Hydrodynamic Radius of Disordered Protein Conformations. Biophys. J..

[85] Kirkwood J.G. (1954). The general theory of irreversible processes in solutions of macromolecules. J. Polym. Sci. Polym. Phys. Ed..

[86] Burchard W. (1999). Solution Properties of Branched Macromolecules. Advances in Polymer Science.

[87] Ohshima H. (2006). Theory of Colloid and Interfacial Electric Phenomena. Interface Science and Technology.

[88] Eslami H., Khani M., Muller-Plathe F. (2019). Gaussian Charge Distributions for Incorporation of Electrostatic Interactions in Dissipative Particle Dynamics: Application to Self-Assembly of Surfactants. J. Chem. Theory Comput..

[89] Delgado A.V., Gonzalez-Caballero F., Hunter R.J., Koopal L.K., Lyklema J. (2005). Measurement and interpretation of electrokinetic phenomena. Pure Appl. Chem..

[90] Wolterink J.K., Leermakers F.A., Fleer G.J., Koopal L.K., Zhulina E.B., Borisov O.V. (1999). Screening in Solutions of Star-Branched Polyelectrolytes. Macromolecules.

[91] Jeffrey G.A., Saenger W. (1991). Hydrogen Bonding in Biological Structures.

[92] Baker E.N., Rossmann M.G., Arnold E. (2006). Hydrogen bonding in biological macromolecules. International Tables for Crystallography Volume F: Crystallography Ofbiological Macromolecules.

[93] Martinho N., Silva L., Florindo H., Brocchini S., Zloh M., Barata T. (2017). Rational design of novel, fluorescent, tagged glutamic acid dendrimers with different terminal groups and in silico analysis of their properties. Int. J. Nanomed..

[94] Luzar A. (2000). Resolving the hydrogen bond dynamics conundrum. J. Chem. Phys..

[95] Balasubramanian S., Pal S., Bagchi B. (2002). Hydrogen-Bond Dynamics near a Micellar Surface: Origin of the Universal Slow Relaxation at Complex Aqueous Interfaces. Phys. Rev. Lett..

[96] Van der Spoel D., van Maaren P.J., Larsson P., Timneanu N. (2006). Thermodynamics of hydrogen bonding in hydrophilic and hydrophobic media. J. Phys. Chem. B.

[97] Darinskii A., Gotlib Y., Lyulin A., Neyelov L.M. (1991). Computer simulation of local dynamics of a polymer chain in the orienting field of the LC type. Vysokomolekularnye Soedineniya. Ser. A.

[98] Kimmich R. (1997). NMR—Tomography, Diffusometry, Relaxometry.

[99] Kruk D., Herrmann A., Rossler E.A. (2012). Field-Cycling NMR Relaxometry of Viscous Liquids and Polymers. Prog. Nucl. Magn. Reson. Spectrosc..

[100] Markelov D.A., Dolgushev M., Gotlib Y.Y., Blumen A. (2014). NMR relaxation of the orientation of single segments in semiflexible dendrimers. J. Chem. Phys..

[101] Markelov D., Dolgushev M., Lahderanta E. (2017). NMR Relaxation in Dendrimers. Annu. Rep. NMR Spectrosc..

[102] Markelov D.A., Fürstenberg F., Dolgushev M. (2018). NMR relaxation in semiflexible Vicsek fractals. Polymer.

[103] Sadovnichy V., Tikhonravov A., Voevodin V., Opanasenko V. (2013). “Lomonosov”: Supercomputing at Moscow State University. Contemporary High Performance Computing: From Petascale toward Exascale.

